# The Efficacy of Periodic Complete Blood Count Tests in Evaluation of the Health Status of Radiation Workers in Iran: A Systematic Review

**Published:** 2020-04

**Authors:** Asma ZARE, Seyed Mohammad Javad MORTAZAVI

**Affiliations:** 1. Department of Occupational Health Engineering, School of Health, Tehran University of Medical Sciences, Tehran, Iran; 2. Department of Medical Physics, School of Medicine, Shiraz University of Medical Sciences, Shiraz, Iran; 3. Department of Diagnostic Imaging, Fox Chase Cancer Center, 333 Cottman Avenue, Philadelphia, PA 19111, USA

**Keywords:** Radiation workers, Complete blood count (CBC), Safety, Risk

## Abstract

**Background:**

Periodic medical examinations of radiation workers are routinely conducted in many countries. Although low dose radiation (LDR) is not expected to cause a significant effect on blood count, the periodic examination usually includes reviewing the work history, general medical history, a physical examination and collecting a blood sample. Despite lymphocytes are the most sensitive cells to radiation, their counts do not show any significant change as long as the radiation level is less than a few hundreds of millisievert (mSv). In spite of this, in Iran, radiation workers, even those who work in diagnostic radiology departments, are regularly examined for blood count changes.

**Methods:**

After a detailed search in PubMed, ISI, Scopus, SID and Google Scholar, only 12 out of 650 articles matched our criteria. A review of these 12 reports was conducted. The full texts were fully reviewed by the authors.

**Results:**

The complete blood count (CBC) test has a very low efficacy in evaluation of the adverse health effects of ionizing radiation in radiation workers. Therefore, finding alternative methods with a higher efficacy is recommended.

**Conclusion:**

CBC tests cannot be introduced as valid markers of potential radiation effects in most occupational exposures. Given this consideration, in periodic tests of radiation workers, cytogenetic tests can be the gold-standard method. In particular, due to its relatively low cost and good sensitivity and specificity, the dicentric assay can be promising. Moreover, the simple and rapid evaluation of micronuclei by fast automated scoring systems can be a good alternative for current low efficacy CBC tests.

## Introduction

One of the most recent advances in technology is the use of ionizing electromagnetic radiation in a wide variety of fields ranging from science to industry and medical applications (1). Ionizing radiation, particularly X-rays and radiations emitted from radioactive materials, play a key role in medical sciences, both in diagnosis and treatment of diseases (2). In spite of this, ionizing radiation is widely known as an occupational hazard in the work environment due to its potential biological damage (3). Exposure to higher levels than the occupational exposure limit (OEL) may cause detrimental biological effects. Ionizing radiation with sufficient energy can damage the molecular structure, resulting in impairment in cell function or mutation. Radiation workers in medical diagnostic and therapeutic centers are inevitably exposed to chronic low-dose ionizing radiation even if they use appropriate personal protective equipment (4). To date, the effects of low doses of ionizing radiation on humans are still controversial (5).

Current findings of cytogenetic studies suggest that long-term exposure to low-doses of ionizing radiation increases the frequency of chromosomal aberrations (6). The frequency of chromosomal injuries in radiation workers - even those exposed to doses below the OEL - was reported to be more than those of the general population (2, 7). The sensitivity of the cells to ionizing radiation varies and hematopoietic cells are the most sensitive cells (8). The number of blood cells in healthy people is relatively constant and changes as a response to factors such as environmental and occupational hazards (9).

CBC is used as a screening test for various blood and non-blood diseases. Despite its simplicity and low cost, CBC plays an important role in the diagnostic and prognostic objectives of the latent period of some diseases, especially chronic ones (9). However, studies performed on the effects of ionizing radiation on blood parameters, show different results. The mean number of the white blood cells, monocytes and plasma immunoglobulin in radiation workers is lower than that of the control group (4, 9). On the other hand, there was no significant difference between the blood indices of radiation workers and the control group (10, 11). Although CBC is now used as a biologic marker of the effects of ionizing radiation on the hematopoietic system, the effectiveness of this method in assessing the effects of ionizing radiation on radiation workers requires more in-depth studies.

This review is an attempt to answer this question whether Complete Blood Count (CBC) is an effective measure for evaluation of the biological effects of radiation in x-ray workers?

## Methods

The present study was conducted as a systematic review of the studies on the effects of exposure to low dose ionizing radiation on blood parameters of Iranian radiation workers. Searching for articles published before between Jan 1, 2000 and March 1, 2019 was performed in the following databases: Scopus, ISI, PubMed, SID, and Google Scholar. After selecting the appropriate articles and rejecting the irrelevant articles and encoding them, analysis of the findings and discussion was carried out.

### Selection Process

Advanced search of studies with the following keywords and their Persian equivalents was done to find the articles published on Complete Blood Count tests in radiation workers (without time limitations in publication date). Radiation worker, radiation field worker, radiation exposure, occupational exposure, ionizing radiation, X-ray, radiology, radiotherapy, nuclear medicine, hematological parameter, blood cells, blood index, blood count, CBC, biological effect, and biological dosimetry were other keywords.

**Fig. 1:**
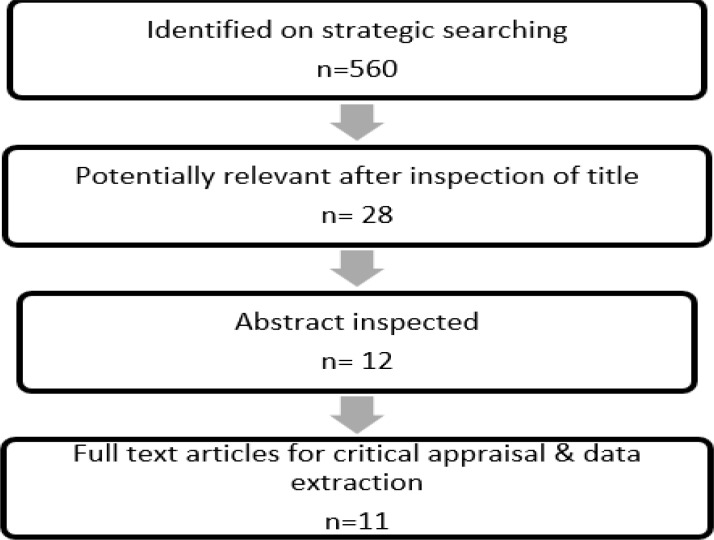
A flowchart demonstrating the selection of articles

Overall, 650 articles in the initial search. The studies conducted in Iran which examined the number of blood cells and related parameters in hospital radiation workers were included. As to selection of the studies, the titles of all selected articles were first checked and the studies conducted outside Iran were excluded. Overall, 28 articles about the effects of radiation on the blood cells (genetic effect on the blood cells, effects on the production of immunoglobulin and effects on the blood cell count) had been carried out on radiation workers in Iran. By reviewing the abstracts, 12 studies in which the blood count of radiation workers was a part of their purpose or the main aim of their work were selected. The blood cells were reviewed in industrial workers (12). In this study, the effect of the whole spectrum of electromagnetic rays on the blood parameters was investigated in a group of welders, furnace workers, and computer users. Not specifying a certain range of radiations (e.g. ionizing radiations), the diversity of occupational groups and the presence of many interfering factors were the reasons for the deletion of this article from the present study. Finally, 11 studies −5 articles in Persian and 6 in English- remained for the review (Fig. 1).

### Encoding articles

In order to do the systematic review of the studies, the articles were classified based on the year of publication, method of research, sample size, the examined parameters, and final results.

## Results

In this section, considering the encoding, the articles were reviewed in different categories.

### Selection Method

All of the studies were case-control type. Case and control groups were selected from the hospital staff. Blood samples were taken from the venous vein and analyzed by using a cell counter system.

### Publication Date Window

**Fig. 2:**
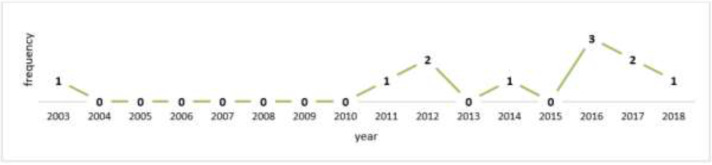
The number of articles based on the year of publication

The articles based on the year of publication are shown in Fig. 2. A study was conducted on hospital radiation workers, but then there was no study until 2011 (13). Most studies were conducted in 2016, followed by two papers in 2012 and two in 2017.

### Population

**Fig. 3:**
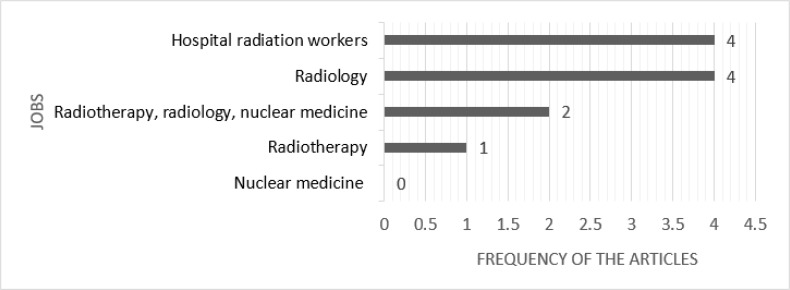
Jobs Investigated in Studies

Four studies had used the general heading of the hospital radiation workers in their study, and it was unclear how many samples were chosen from the radiology, radiotherapy or nuclear medicine sections (9, 13–15). Two studies were conducted on radiologists, radiotherapists and nuclear medicine, with a specific number of samples from each section (Fig. 3) (16, 17). Of all the studies, four were performed on radiologists (10, 11, 18, 19) and one on radiotherapists (20). In general, the highest number of studies was done on radiologists.

### Sample Size

**Fig. 4:**
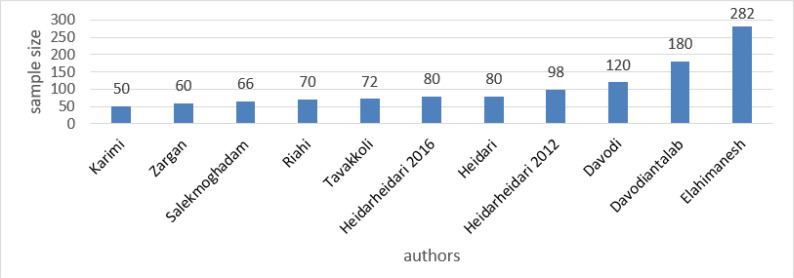
Sample size in evaluated studies

In Fig. 4, the sample size of the studies is shown. The highest number of samples was studied in Elahimanesh et al. (15), Davoudiantalab et al. (10) and Davoudi et al. (20). The lowest number of samples was also observed in Salek Moghaddam et al. (13), Zargan et al. (14) and Karimi et al. (11).

### Studied Parameters

The examined blood parameters were different in the studies considered (Table 1). As outlined in Table 1, the number of white blood cells (WBCs) was studied in all 11 studies, followed by the platelets and RBC count in10 studies. Among the studies, Riahi et al. (18) and Heidari et al.(19) the highest number of parameters (15 parameters) was examined (18,19), and Salek Moghaddam et al.(13) had the least number of parameters (2 parameters). The ionizing radiation in 27% of the cases had a significant effect on the number of white blood cells (3 of 11 studies). The number of lymphocytes in 2 out of 9 studies and the number of platelets in 2 out of 10 studies showed a significant effect from exposure to ionizing radiation. Ionizing radiation in any of the studies did not have a significant effect on the parameters associated with the red blood cells.

**Table 1:** Blood parameters in the studies

***Studies*** ***Parameters***	***Salek Moghaddam***	***Davodiantalab***	***Karimi***	***Elahimanesh***	***Heidari***	***Heidarheidari2016***	***Zargan Riahi***		***Davodi***	***Heidarheidari 2012 (16)***	***tavakoli***	***Total***	***Number of significant parameters***
WBC	^*^	^*^	^*^	^*^	^*^ (*P*=0.013)	^*^	^*^	^*^	^*^ (*P*<0.05)	^*^	^*^ (*P*=0.002)	11	3
Platelet		^*^	^*^	^*^	^*^ (*P*=0.041)	^*^	^*^	^*^	^*^ *(P*<0.01)	^*^	^*^	10	2
Lymphocyte	^*^	^*^ (*P*=0.04)	^*^	^*^	^*^ (*P*=0.034)	^*^		^*^		^*^	^*^	9	2
Hemoglobin		^*^	^*^	^*^	^*^	^*^	^*^	^*^	^*^	^*^		9	0
MCV					^*^	^*^		^*^	^*^	^*^		5	0
RBC		^*^	^*^	^*^	^*^	^*^	^*^	^*^	^*^	^*^	^*^	10	0
PDW					^*^			^*^ (*P*=0.028)				2	1
PLC-R								^*^ (*P*=0.015)				1	1
Neutrophil		^*^	^*^	^*^	^*^ (*P*=0.034)	^*^		^*^		^*^	^*^	8	1
Monocyte		^*^			^*^	^*^				^*^	^*^ *P*=0.000)	5	1
Hematocrit		^*^	^*^	^*^	^*^	^*^	^*^	^*^		^*^		8	0
MPV					^*^			^*^				2	0
MCHC					^*^			^*^	^*^			3	0
MCH					^*^			^*^	^*^			3	0
RDW								^*^	^*^			2	0
Basophil					^*^					^*^		2	0
Eosinophil		^*^			^*^					^*^	^*^	4	0
Reticulocyte								^*^				1	0
Total	2	9	7	7	15	9	5	15	8	11	7	2	

### Outcomes

Five out of 11 studies did not find any significant effect of ionizing radiation on the number of blood cells and confirmed that low dose ionizing radiation did not have a significant effect on the number of blood cells (10, 11, 13, 14, 16). Six other studies observed significant changes in one or more parameters; some studies have suggested that ionizing radiation has an effect on the blood cell count (9, 17, 18), and some studies have justified their observations and rejected the effect of radiation on the blood cell count (15, 19, 20). Heidarheidari et al. (17) and Elahimanesh et al. (15) observed the significant effect of ionizing radiation on the number of blood cells in individuals with a working experience of more than 10 years in one or two parameters. In general, 8 out of 11 studies reported that low-dose ionizing radiation had no effect on blood-counting (Table 2).

**Table 2:** The final outcome of the studies evaluated

***Outcome Studies***	***Significant effects of ionizing radiation on blood parameters were NOT observed.***	***The assumption of no effect of radiation on the number of blood parameters is approved***
Salek Moghadam (13)	^*^	^*^
Tavakoli (9)		
Heidarheidari 2012 (16)	^*^	^*^
Davodi (20)		^*^
Riahi (18)		
Zargan (14)	^*^	^*^
Heidarheidari 2016 (17)		
Heidari (19)		^*^
Elahimanesh (15)		^*^
Karimi (11)	^*^	^*^
Davodiantalab (10)	^*^	^*^
Total	5	8

## Discussion

Hospital radiation workers are always exposed to low doses of ionizing radiation. Therefore, the risk assessment of the factors associated with this exposure is required. In Iran, regular periodic CBC tests are performed to evaluate the health of the radiation workers. In this study, the validity of the CBC test in assessing the effects of ionizing radiation on the blood was evaluated. Based on the findings of our study, there were only a very limited number of scientific reports in this field and these reports had controversial results. Our findings showed no effect of low-dose ionizing radiation on the parameters linked to red blood cells (RBC) such as Hematocrit, MCV, MCH, MCHC, and RDW) and hemoglobin. Red blood cells did not show any significant changes after occupational exposure to low doses of radiation (21, 22).

Different results were observed for parameters linked to white blood cells (WBC) such as the counts of Lymphocytes, Neutrophils, and Monocytes as well as platelets (Platelet, PDW, PLC-R). As indicated in Table 1, researchers observed significant changes in the blood cells associated with the immune system (9, 15, 18–20). This finding can be due to the selection of the control group in these studies. Interestingly, in all of these five studies, the control group was selected from other departments of the hospitals (Non-radiation Departments). The staff of different departments of any hospital is continuously exposed to various types of pathogens and, therefore, their immune system has become stronger over time. Mean T cells of hospital staff were compared with the normal values indicated in the kit as well as the control groups in previous studies and found that the blood cells associated with the immune system did not show a drop in radiation workers. However, the active T cells of the radiation workers were higher than the general population (13). Therefore, choosing the control group from different parts of the hospital exposed to various infectious agents can lead to invalid results. On the other hand, the significant effect of ionizing radiation on the blood parameters was considered as a response to the increase of work experience (15,21). However, in other studies, no relationship between this parameter and increased work experience was found. Alteration of some blood parameters as a response to the increased accumulated exposure to low doses of radiation due to higher work experience, can be associated with higher age in experienced workers (13, 23). Despite limitations of CBC as a valid medical examination of radiation workers, it has some advantages other than speed, simplicity and low cost. For example, thrombocytopenia, anemia and neutropenia are sometimes the first signs of hematologic malignancies In this case, CBC plays a key role in diagnostic algorithm of thrombocytopenia (24).

The key limitations of the studies included in our study are the lack of measurement of the dose rate as well as lack of accurate dose reporting system. Only Heidarheidari et al. reported the exact dose of radiation received by radiation workers (17). In other studies, it was only indicated that exposure to ionizing radiation was less than the dose limit. On the other hand, the sample size in the majority of studies was below 100 radiation workers, and almost 50% of these participants were control group. Therefore, the number of participants in all of these studies was very low. Another important issue that amplifies the problem of small sample size, is the wide variations in genetic susceptibility of different individuals to ionizing radiation. While genetic factors have a significant effect on the sensitivity of individuals to radiation (25), the failure to consider these variations as a basic confounding factor, in particular when the sample size is small, can affect the validity of the results.

Given this consideration, altogether, CBC tests cannot be introduced as valid markers of potential radiation effects in most occupational exposures, particularly in occupational exposure settings with low annual radiation doses such as x-ray departments. In a similar pattern with biological dosimetry, in periodic tests of radiation workers, cytogenetic tests can be introduced as the “gold-standard” method. In particular, due to its specificity, the dicentric assay (Dic) in peripheral blood lymphocytes of radiation workers (PBL) can be promising (26). When any overexposure is likely, the dicentric assay is still the gold standard biodosimetry method due to its highly standardized method for evaluation of individual doses (27). Moreover, using a dose-response calibration curve for dicentrics, biodosimetry would be possible(28). Moreover, the evaluation of micronuclei (MN) by fast automated scoring systems can be a good alternative for current low efficacy CBC tests.

In South Korea, the clinical usefulness of CBC tests for radiation workers was investigated. The radiation absorbed doses calculated by cytogenetic biodosimetry, all the consecutive results of CBC, as well as the TLD doses, were studied. For health protection regulation, CBC tests and cytogenetic studies are useful complementary tools to TLD doses (29). In Croatia, the cytogenetic effects of low doses of x-rays in blood lymphocyte cultures of 765 hospital staff occupationally exposed to different sources of chromosome damaging agents was compared with 200 control subjects. Only urologists/gynaecologists showed statistically significant higher levels of dicentric chromosomes. These findings clearly showed the importance of periodic medical checkups of health workers occupationally exposed to low dose radiation (LDR)(30).

In Iran, both MN assay (31) and Dic (32) have been widely used in different departments. In addition, automated systems can efficiently increase the speed of Dic and MN in peripheral blood lymphocytes of radiation workers. However, the key disadvantage of automated systems is their high cost for departments in developing countries. The second limitation of automated systems is the lower number of dicentrics identified by automated systems. It spite of this, these limitations can be, at least to some extent, partially compensated by the higher speed of metaphase finding (33).

## Conclusion

Altogether, chronic occupational exposure to low doses of ionizing radiation has no significant effect on the blood count parameters. In this study, even lymphocytes, as the most sensitive cells, did not show a significant response to radiation at ordinary occupational levels. Given this consideration 1) As long as the exposure levels are low and proper radiation protection methods are used by the personnel, there is no significant risk for radiation workers. 2) If we are looking for medical examination of radiation workers with higher levels of efficacy, replacing current CBC examinations with other methods seems to be justified. Generally, CBC tests, at least for some types of occupational exposures with low annual radiation doses (e.g. x-ray technologists) has very low efficacy in detecting the detrimental effects of ionizing radiation. Thus, finding alternative methods with higher sensitivity is strongly recommended. The frequency of chromosome aberrations in radiation workers is higher than the general population (not exposed to occupational sources of radiation). The traditional evaluation of chromosome aberrations or micronuclei, or using fast automated scoring systems can be good alternatives for current low efficacy CBC tests.

## Ethical considerations

Ethical issues (Including plagiarism, informed consent, misconduct, data fabrication and/or falsification, double publication and/or submission, redundancy, etc.) have been completely observed by the authors.
